# A case of unusually persistent traumatic intrapulmonary hematoma

**DOI:** 10.1016/j.rmcr.2021.101507

**Published:** 2021-09-03

**Authors:** Sachi Kawagishi, Yuya Kogita, Itsuko Nakamichi, Takahiro Matsui, Toshiteru Tokunaga

**Affiliations:** a Department of General Thoracic Surgery, Minoh City Hospital, 5-7-1, Kayano, Minoh, Osaka, 562-0014, Japan; b Department of General Thoracic Surgery, National Hospital Organization Osaka Toneyama Medical Center, 5-1-1, Toneyama, Toyonaka, Osaka, 560-8552, Japan; c Department of Pathology, Minoh City Hospital, 5-7-1, Kayano, Minoh, Osaka, 562-0014, Japan; d Department of Pathology, Osaka University Graduate School of Medicine, 2-2, Yamadaoka Suita, Osaka, 565-0871, Japan

**Keywords:** Intrapulmonary hematoma, Surgical resection, Chest injury, CT, computerized tomography, FDG-PET, 18F-fluorodexyglycose positron emission tomography, MRI, Magnetic resonance imaging

## Abstract

Intrapulmonary hematomas are collections of blood within alveolar and interstitial spaces. They occur mainly following thoracic trauma. Typically, intrapulmonary hematomas without bleeding or infection spontaneously disappear after several weeks to 6 months. In the current case, the patient presented with an intrapulmonary nodule 17 months after a chest injury. The size of the nodule had not changed at 4 months after the first visit. Consequently, the patient was diagnosed with an intrapulmonary hematoma by surgical resection. To our knowledge, there are no previous studies that described the cause of the persistent intrapulmonary hematoma. This study reports the case of a patient who underwent surgical resection of a persistent traumatic intrapulmonary hematoma.

## Highlights

• Intrapulmonary hematoma occurs with a chest injury, anticoagulants, thrombocytopenia, and congenital diseases.
• Differential diagnoses should include intrapulmonary hematoma when considering medical history.
• Patients are generally placed under observation when intrapulmonary hematoma is suspected.
• This is the first report on the cause of persistent intrapulmonary hematoma.

## 1 Introduction

Intrapulmonary hematomas are collections of blood within the alveolar and interstitial spaces. Typically, they spontaneously disappear from several weeks to 6 months.[1,2] In the current case, the patient presented with an intrapulmonary nodule 17 months after a chest injury. The nodule's size had not changed at 4 months after the first visit, and the patient was diagnosed with intrapulmonary hematoma via surgical resection. To our knowledge, this is the first report that describes the etiology of a persistent intrapulmonary hematoma. Here, we present the case of a patient who underwent surgical resection of a traumatic intrapulmonary hematoma that did not disappear after a long follow-up period.

## 2 Case report

**Fig. 1:**
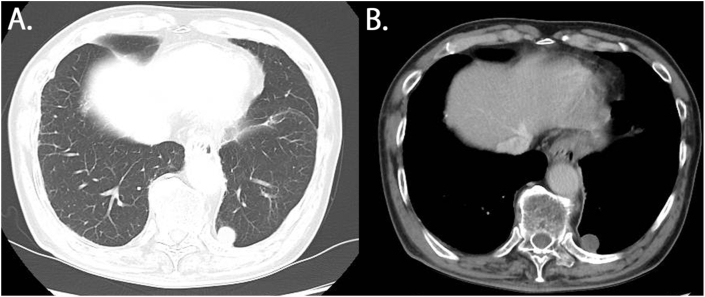
Chest computerized tomography findings. **A** The nodule is located at the side of the left lower lobe or posterior mediastinum. **B** The nodule showed no contrast effect.

Chest X-ray findings during a medical checkup of a 70-year-old man showed an abnormal shadow. Chest computed tomography (CT) revealed a nodule on the side of the left lower lobe or posterior mediastinum (Fig. 1A). The patient's medical history showed that he had multiple rib fractures and traumatic left hemopneumothorax due to falls 17 months before the first visit to our hospital. In addition, he had undergone chest tube insertion at another hospital. Three days after insertion of that tube, there was no leakage, and the amount of daily drainage was decreasing. Chest X-ray revealed improvement in the left hemothorax and the chest tube was removed. At this point, he had evidence of left lower pulmonary lobe contusion; however, no nodule was identified.

**Fig. 2:**
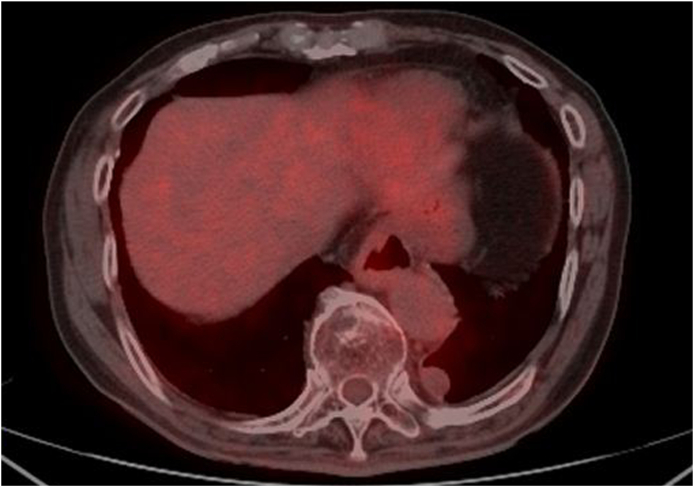
18F-fluorodexyglycose positron emission tomography/computerized tomography revealed uptake (the maximum standardized uptake value = 1.66) in the lesion.

Contrast-enhanced chest CT of our hospital revealed a nodule in the left lower lobe (S10). The nodule was 27 × 18 × 16 mm in size and was elliptical with well-defined margins. It showed no contrast effect (Fig. 1B). Furthermore, 18F-fluorodexyglucose positron emission tomography/CT (FDG-PET/CT) revealed a little uptake (the maximum standardized uptake value = 1.66) in the lesion (Fig. 2). Chest CT performed 4 months after the first visit showed that nodule size had not changed. The patient had no history of malignancy, making a metastatic tumor unlikely. Taking the shape of the nodule and his history together, we suggested that the differential diagnosis was a benign lung tumor, a neurogenic tumor in the posterior mediastinum, a low-grade malignant tumor such as a pulmonary carcinoid. The resection surgery for diagnostic treatment was planned to rule out a malignant tumor.

**Fig. 3:**
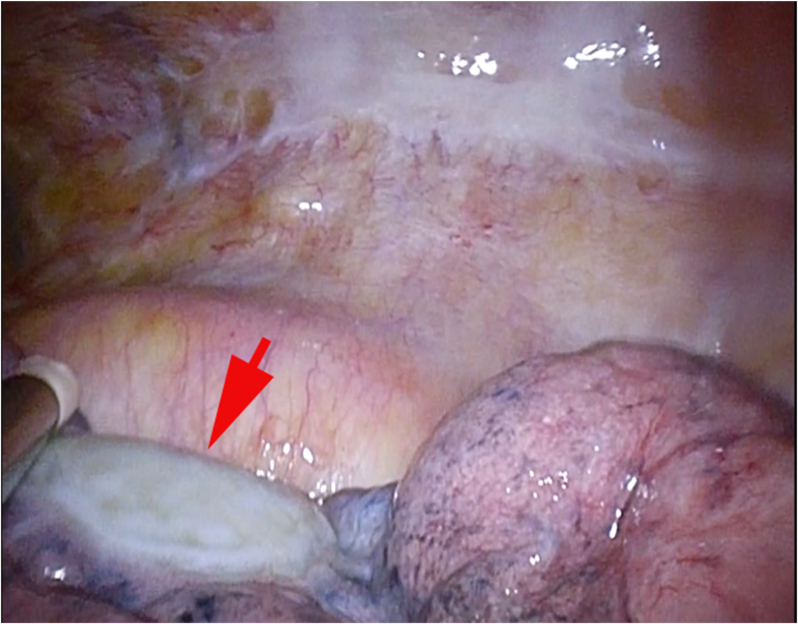
Intraoperative picture of the lesion. The white pleural change located at the left lower lobe (arrow).

Partial resection of the left lower lobe was performed by thoracoscopic surgery. Intrathoracically, the white pleural change was at the left lower lobe. This pleural change was considered as the nodule that was visualized in the image, and a partial resection was performed (Fig. 3). The intraoperative pathological diagnosis was not a malignant finding, and only a partial resection was performed. The divided surface of the nodule showed a dark-red pasty material. The operation time was 84 minutes, and the amount of blood loss was 10 ml. The postoperative course was good, and the patient was discharged 6 days after the procedure.

**Fig. 4:**
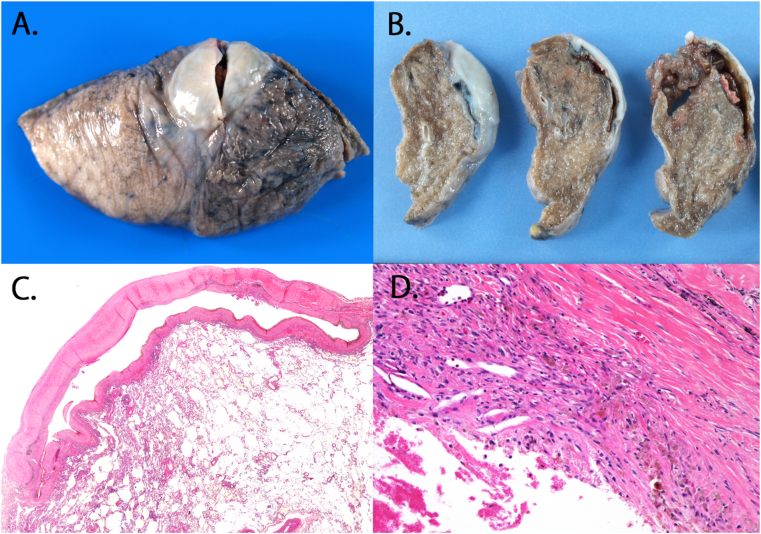
**A**, **B** Specimen findings. A part of the white pleural change had gross pleural thickening and cyst-like features located directly below the pleural change. **C** Hematoxylin and eosin staining of the nodule. The area of the grayish-white capsule had a cyst-like features that was responsible for the hyalinization of connective tissue (magnification × 40). **D** The inside of the cyst was composed of red cells, fibrin, and hemosiderin-laden macrophage (magnification × 100). (For interpretation of the references to colour in this figure legend, the reader is referred to the Web version of this article.)

In the specimen, a portion of the white pleural change had gross pleural thickening and had cyst-like features located directly below the pleural change. Postoperative pathological findings revealed that the area of the grayish-white capsule showed cyst-like structures and was responsible for the hyalinization of the connective tissue. In high-power fields, the inside of the cyst was filled with red blood cells, fibrin, and hemosiderin-laden macrophage. Considering this, it was our understanding that the nodule was a collection of blood (Fig. 4).

Here were present the case of a patient who had a medical history of chest injury and was diagnosed with traumatic intrapulmonary hematoma.

## 3 Discussion

Intrapulmonary hematomas are collections of blood within the alveolar and interstitial spaces [1]. The most common etiology of intrapulmonary hematoma is considered to be thoracic injury; specifically, an external force that has been transferred through the thoracic wall to the lungs, directly below the pleura. The bleeding observed inside the pulmonary alveolus or the interstitial space was caused by damage to the structure of the capillary blood vessels of the pulmonary alveolus. However, cases of intrapulmonary hematoma with no history of trauma are reported, and such cases are referred to as spontaneous pulmonary hematoma. Spontaneous pulmonary hematomas are extremely rare and have been confirmed to occur with anticoagulant therapy, thrombocytopenia, and congenital diseases such as Ehlers–Danlos syndrome.[3,4] In addition, Lee reported that cases of intrapulmonary hematomas of an unknown etiology were very rare [1].

According to previous reports, intrapulmonary hematomas without bleeding or infection usually disappear spontaneously after several weeks to 6 months [2]. Chon reported that traumatic intrapulmonary hematomas disappear after a mean period of 145.8 days [5]. On the other hand, Okada et al. reported a solitary, 3-cm-diameter, round nodule that initially was not treated and was diagnosed as an expanding hematoma by resection 7 years later [6]. The hematoma in our case, which did not disappear after 17 months following a chest injury, was similarly unusual.

Chest X-ray and chest CT of the intrapulmonary hematoma showed a well-circumscribed and inhomogeneous round nodule; however, these findings were not specific [7]. According to previous studies, magnetic resonance imaging shows a hyperintense mass on T1 and T2-weighted imaging, and it can be useful for the differential diagnosis between a intrapulmonary hematoma and a malignant tumor [8]. In cases when intrapulmonary hematoma is suspected through the patient's medical history and imaging outcomes, the patient is generally placed under observation. Nodules diagnosed as intrapulmonary hematomas by means other than resection, such as percutaneous biopsy or bronchoscopy, should be followed up because the possibility of an expanding hematoma remains [6]. Nearly all cases of intrapulmonary hematoma disappear during the follow-up period [2]. If no diagnosis is established for a pulmonary nodule, other investigations should be initiated during follow-up. Increase in size, uptake of 18F-fluorodexyglucose in positron emission tomography/CT images, or a contrast effect in contrast-enhanced chest CT are all indicators of the possibility of a malignant tumor, in which case resection for diagnostic and treatment purposes should be planned.

Although the underlying mechanism of expanding hematomas remains unknown, it is often explained though the mechanism of chronic epidural hematomas. In this report, the chronic hematoma shows the formation of a blood-filled capsule caused by a chest trauma. Inflammation under this capsule and the presence of the cellular breakdown products of leukocytes, erythrocytes, platelets, and fibrin cause further damage and bleeding from capillaries. It has been speculated that this mechanism is responsible for the expansion, and not the attenuation, of hematomas [9]. However, almost all cases of intrapulmonary hematomas have minimal inflammation and do not cause damage to the capillaries. The blood in the capsule is broken down and absorbed by hemosiderin, and the capsule eventually disappears.

Regarding the pathology of our case, we confirmed the presence of a cyst-like structure with hyalinized connective tissue around the lesion, along with thick walls consisting of rigid and dense connective tissue, localized directly under the pleura. A large number of capillary blood vessels were present in the hyalinized connective tissue on the distal side of the pleura of the lesion, wherein the lesion was accompanied by the infiltration of inflammatory cells. A clotted mass with an intact fibrin net was present despite the collapse of the red blood cells and the morphology of blood cells inside the lesion. Moreover, we also noted that the red blood cells in the lesion were phagocytosed by the histiocytes as hemosiderin. During our observation of the structure of the capillary blood vessels, we noted that the process of the breakdown of red blood cells into hemosiderin and its subsequent absorption could be attributed to chronic inflammation and immediate bleeding caused by trauma inside the lesion. Conversely, the distribution of the capillary blood vessels inside the rigid and dense connective tissue directly below the pleura was sparse. Therefore, lack of absorption had progressed in this area. The stage at which this rigid and dense connective tissue formed directly below the pleura was pathologically unclear. Thus, it is possible that incidental bleeding occurred directly under the area where the connective tissue was thick, or some other etiology was responsible for the causing the formation of thick connective tissue specifically in the region near the pleura. From these findings, it can be inferred that a balance between bleeding and absorption in the hematoma could be caused the persistence of the hematoma without being absorbed after the trauma. To our knowledge, this is the first reported case to simultaneously show evidence of ongoing bleeding and absorption. We consider that these findings exclude the diagnosis of expanding hematoma.

This was a case of an intrapulmonary hematoma that was accidently identified via an imaging examination from a medical checkup. It would not have been possible to eliminate the possibility of an expanding hematoma if we had not excised the lesion. After performing surgical resection of the traumatic intrapulmonary hematoma, the recurrence rate of intrapulmonary hematoma is considered low if the patient does not undergo anticoagulant therapy. Therefore, a long-term follow-up is not needed.

## 4 Conclusion

We surgically resected a traumatic intrapulmonary hematoma that did not disappear after a long follow-up period. Pathologically, a balance of bleeding and absorption in the hematoma caused the hematoma to persist without being absorbed after the trauma. Some intrapulmonary hematomas progress in an unusual manner, as observed in this case. It is necessary to consider intrapulmonary hematoma in differential diagnosis by taking into account the patient's medical history. Furthermore, diagnosis through imaging examinations is difficult, and biopsy or surgical resection should be considered.

## Author contributions

Sachi Kawagishi: Writing - Original Draft, Yuya Kogita: Validation, Itsuko Nakamichi: Resources, Takahiro Matsui: Resources, Toshiteru Tokunaga: Writing - Review & Editing, Supervision.

## Funding

This research did not receive any specific grant from funding agencies in the public, commercial, or not-for-profit sectors.

## Declaration of competing interest

The authors declare that they have no known competing financial interests or personal relationships that could have appeared to influence the work reported in this paper.
